# Atorvastatin‐Associated Severe Bradyarrhythmia Following Multiorgan Dysfunction: A Case Report

**DOI:** 10.1002/ccr3.73270

**Published:** 2026-07-31

**Authors:** Xiao‐Qing Kou, Yan‐Zhen Wang, Yi‐Rong Gan, Yun‐Long Zhang, Shu‐Yun He, Zheng Zhang

**Affiliations:** ^1^ Institute of Cardiovascular Diseases Lanzhou First People's Hospital Lanzhou Gansu Province China; ^2^ The First School of Clinical Medicine of Lanzhou University Lanzhou Gansu Province China; ^3^ Department of Cardiology The First Hospital of Lanzhou University Lanzhou Gansu Province China

**Keywords:** atorvastatin, bradyarrhythmia, multiorgan dysfunction, pacemaker, sinus arrest, sinus node dysfunction

## Abstract

Statins are the primary treatment for hypercholesterolemia and a cornerstone of atherosclerotic cardiovascular disease prevention. Although generally safe, serious adverse effects may occur in selected patients, particularly when statins are used at high doses or without medical supervision. We describe a 66‐year‐old man with hypertension and hyperlipidemia who developed syncope after self‐adjusting atorvastatin from 20 mg daily to 80 mg daily for two weeks. On admission, electrocardiography showed sinus arrest with a ventricular escape rhythm, accompanied by acute hepatic and renal dysfunction, electrolyte abnormalities, coagulation abnormalities, and elevated inflammatory markers. Common reversible causes, including thyroid dysfunction, structural heart disease, acute myocarditis, Lyme disease, and the use of negative chronotropic drugs, were not supported by the clinical evaluation. After temporary pacing and supportive treatment, liver and kidney function, electrolytes, and coagulation function returned to normal; however, symptomatic sinus arrest persisted, and a permanent pacemaker was implanted. During follow‐up, the electrocardiogram showed recovery of sinus rhythm. Pacemaker interrogation at 1 year showed a pacing burden of approximately 80% when the lower rate limit was programmed at 70 beats per minute; after the lower rate limit was reduced to 50 beats per minute, the pacing burden decreased to approximately 20% at the subsequent follow‐up. This finding suggests partial recovery of intrinsic sinus rhythm but persistent or intermittent sinus node dysfunction. The case supports a cautious interpretation of possible atorvastatin‐associated severe bradyarrhythmia mediated by multiorgan dysfunction rather than definitive direct statin‐induced sinus node injury. Clinicians should carefully educate patients not to self‐adjust statin doses and should monitor for serious adverse events when high‐dose statin exposure is suspected.

**Abbreviations**
ALT: alanine aminotransferase
AST: aspartate aminotransferase
CK‐MB: creatine kinase‐myocardial band
CRP: C‐reactive protein
cTnI: cardiac troponin I
ECG: electrocardiogram
FDP: fibrin degradation products
FT3: free triiodothyronine
FT4: free thyroxine
hs‐CRP: high‐sensitivity C‐reactive protein
LDL‐C: low‐density lipoprotein cholesterol
LRL: lower rate limit
MDRD‐eGFR: estimated glomerular filtration rate calculated using the Modification of Diet in Renal Disease equation
MYO: myoglobin
NT‐proBNP: N‐terminal pro‐B‐type natriuretic peptide
PT: prothrombin time
TCHO: total cholesterol
TPOAb: anti‐thyroid peroxidase antibody
TSH: thyroid‐stimulating hormone
WBC: white blood cell

**Key Clinical Message**

Severe bradyarrhythmia may occur in the setting of unsupervised high‐dose atorvastatin exposure complicated by acute hepatic and renal dysfunction, electrolyte imbalance, inflammation, and metabolic disturbances. Clinicians should carefully review statin dosing history, correct reversible abnormalities, and recognize that persistent or intermittent sinus node dysfunction may require temporary or permanent pacing support and long‐term follow‐up.

## 1 Introduction

Statins inhibit 3‐hydroxy‐3‐methylglutaryl coenzyme A reductase and are first‐line therapies for lowering low‐density lipoprotein cholesterol and preventing atherosclerotic cardiovascular events [1]. Their overall safety profile is favorable, and serious muscle or hepatic injury is uncommon [2]; nevertheless, adverse effects may become clinically important with high‐dose exposure, drug interactions, organ dysfunction, or inappropriate self‐adjustment of therapy [3].

Cardiac conduction disturbances are not recognized as common or characteristic adverse effects of statin therapy, and direct clinical evidence linking atorvastatin to sinus node dysfunction remains limited [4]. This case is clinically distinctive because profound sinus arrest occurred together with acute hepatic and renal dysfunction and major metabolic abnormalities, persisted after the reversible systemic abnormalities improved, and subsequently showed partial recovery on long‐term pacemaker interrogation. The objective of this report is to describe the diagnostic reasoning, treatment, and follow‐up while avoiding an unsupported claim of direct drug causality.

## 2 Case History

The patient, a 66‐year‐old male, had a history of hypertension and was diagnosed with hyperlipidemia. His antihypertensive regimen consisted of telmisartan 40 mg once daily, with no history of calcium channel blockers, beta‐blockers, digoxin, antiarrhythmic drugs, or other negative chronotropic drug use. He also reported no use of ayurvedic, herbal, or other non‐prescribed medications for hypertension or dyslipidemia. Two months prior, a routine electrocardiogram (ECG) showed sinus rhythm and a normal ECG (Figure 1). Initially prescribed atorvastatin 20 mg daily, the patient increased the dose to 80 mg daily without medical supervision in an attempt to rapidly lower his lipid levels. Shortly after this self‐adjustment, he began experiencing dizziness and fatigue, which he initially ignored. As his symptoms worsened, he developed intermittent visual disturbances, eventually leading to syncope. Upon admission, the patient's blood pressure was 108/58 mmHg, and his heart rate was 35 beats per minute. ECG revealed sinus arrest and a ventricular escape rhythm (Figure 2).

**FIGURE 1:**
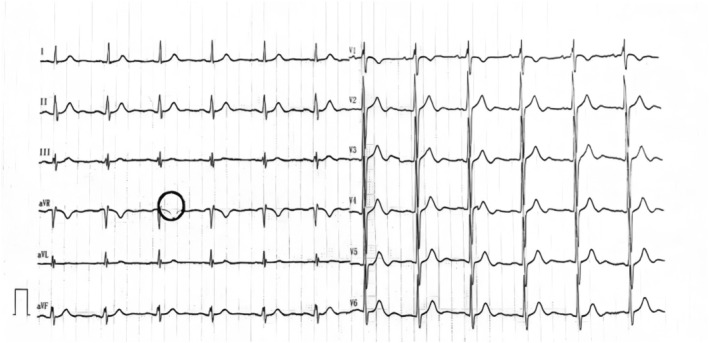
Baseline ECG performed two months before admission, showing sinus rhythm and a normal ECG.

**FIGURE 2:**
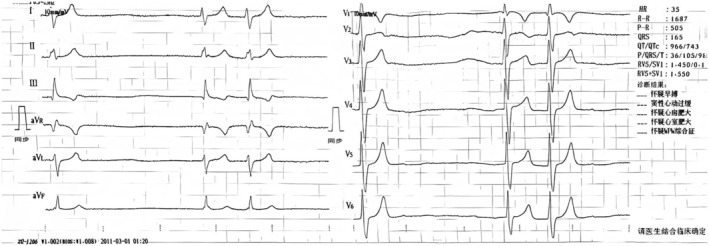
Admission ECG after syncope, showing sinus arrest and a ventricular escape rhythm.

## 3 Methods (Differential Diagnosis, Investigations and Treatment)

A temporary cardiac pacemaker was implanted. Laboratory tests revealed the following results: N‐terminal pro‐B‐type natriuretic peptide (NT‐proBNP) 1912 ng/L (reference range: 0–900), creatine kinase‐myocardial band (CK‐MB) < 2.5 ng/mL (reference range: 0.0–5.0), myoglobin (MYO) 213 μg/L (reference range: 0–70), cardiac troponin I(cTnI) < 0.01 ng/mL (reference range: 0.00–1.00), urea 22.0 mmol/L (reference range: 3.6–9.5), creatinine 405 μmol/L (reference range: 57–111), estimated glomerular filtration rate calculated using the Modification of Diet in Renal Disease equation (MDRD‐eGFR) 14 mL/min/1.73m^2^ (reference range: > 90), alanine aminotransferase (ALT) 2107 U/L (reference range: 9–50), aspartate aminotransferase (AST) 3585 U/L (reference range: 15–40), white blood cell (WBC) 12.9 × 10^9^/L (reference range: 3.5–9.5 × 10^9^/L), C‐reactive protein (CRP) 26.70 mg/L (reference range: 0.00–10.0), high‐sensitivity C‐reactive protein (hs‐CRP) > 5.0 mg/L (reference range: 0.00–3.00), prothrombin time (PT) 18.6 s (reference range: 10.4–12.6), fibrin degradation products (FDP) 19.0 μg/mL (reference range: 0.0–5.0), D‐Dimer 3.92 μg/mL (reference range: 0.00–0.50), total cholesterol (TCHO) 1.56 mmol/L (reference range: 3.89–5.17), low‐density lipoprotein cholesterol (LDL‐C) 0.61 mmol/L (reference range: 2.06–3.10), potassium 4.79 mmol/L (reference range: 3.50–5.30), sodium 121.1 mmol/L (reference range: 137.0–147.0), chloride 86.1 mmol/L (reference range:99.0–110.0), calcium 1.87 mmol/L (reference range: 2.11–2.52). thyroid function tests were within the reference range: free triiodothyronine (FT3) 5.94 pmol/L (reference range: 3.09–6.71), free thyroxine (FT4) 17.99 pmol/L (reference range: 12.00–22.00), thyroid‐stimulating hormone (TSH) 2.865 mIU/L(reference range: 0.271–4.21), and anti‐thyroid peroxidase antibody (TPOAb) 23.61 IU/mL (reference range: 0.0–30.20). Cardiac ultrasound revealed no structural abnormality or evidence suggestive of infiltrative cardiomyopathy. There was no clinical evidence suggestive of Lyme disease or acute myocarditis, although Lyme serology was not performed. The normal cTnI and CK‐MB values did not support acute myocardial cell injury. the isolated myoglobin elevation was considered nonspecific in the setting of severe renal dysfunction and possible skeletal‐muscle release.

The differential diagnosis included medication‐associated bradyarrhythmia, metabolic and electrolyte disturbance‐related sinus node dysfunction, ischemic or inflammatory myocardial disease, thyroid disease, structural cardiac disease, and other drug‐induced bradyarrhythmia. The temporal relationship to high‐dose atorvastatin exposure, the presence of multiorgan dysfunction, and the absence of alternative common causes supported a cautious diagnosis of possible atorvastatin‐associated severe bradyarrhythmia mediated by systemic illness.

Atorvastatin was discontinued. Treatment included temporary pacing, correction of electrolyte abnormalities, fresh frozen plasma, reduced glutathione, and supportive treatment aimed at improving hepatic, renal, and coagulation function. After one week, liver and kidney function, electrolytes, and coagulation parameters had substantially improved. However, repeat ECG continued to show sinus arrest and a ventricular escape rhythm (Figure 3) and symptomatic episodes persisted. Because clinically significant sinus node dysfunction remained despite correction of reversible abnormalities, a permanent dual‐chamber pacemaker was implanted after shared decision‐making and written informed consent (Figure 4).

**FIGURE 3:**
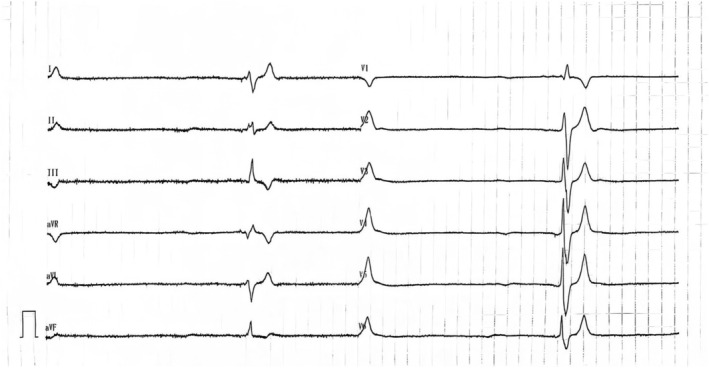
ECG after one week of supportive treatment showing persistent sinus arrest with a ventricular escape rhythm.

**FIGURE 4:**
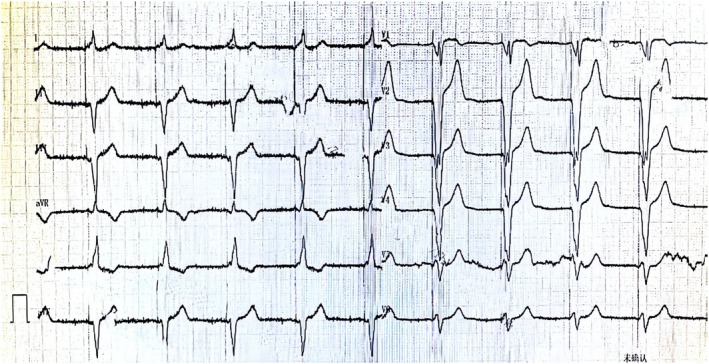
ECG after implantation of a permanent dual‐chamber pacemaker showing an appropriately paced rhythm.

## 4 Conclusions and Results (Outcome and Follow‐Up)

The patient was discharged in stable condition and scheduled for outpatient follow‐up. Post‐discharge evaluations of liver function, renal function, and coagulation function were unremarkable. ECG after permanent pacemaker implantation showed a paced rhythm with normal pacing and sensing function (Figure 4). After 1 year of follow‐up, the ECG showed sinus rhythm (Figure 5). Pacemaker interrogation showed VAT mode pacing with a pacing burden of approximately 80% when the lower rate limit (LRL) was set to 70 beats per minute. After the LRL was adjusted to 50 beats per minute, interrogation at the subsequent 1‐month follow‐up revealed a reduction in pacing burden to approximately 20%. This reduction indicates that the initial high burden was partly programming dependent and that intrinsic sinus rhythm had partially recovered, although persistent or intermittent sinus node dysfunction remained.

**FIGURE 5:**
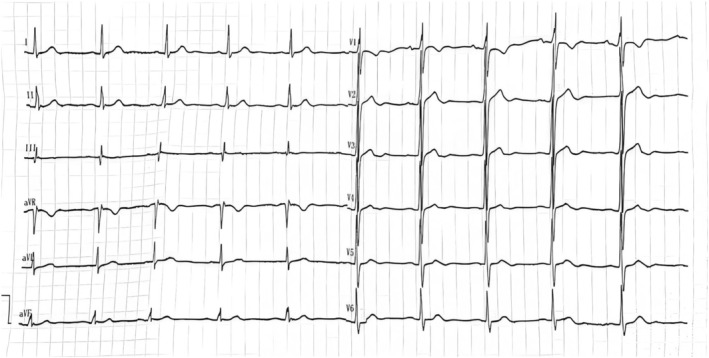
One‐year follow‐up ECG showing recovery of sinus rhythm.

## 5 Discussion

This case demonstrates a temporal association between unsupervised high‐dose atorvastatin exposure, acute multiorgan dysfunction, and severe sinus node dysfunction. It does not establish direct atorvastatin cardiotoxicity. Large randomized trials, meta‐analyses, and scientific statements support the overall cardiovascular efficacy and favorable safety profile of statins [3, 5]. Although statins generally have a strong safety profile, some patients may experience adverse reactions. The reported prevalence of statin intolerance varies widely, ranging from approximately 5% in randomized controlled trials to as high as 30% in observational studies; a large meta‐analysis estimated a pooled prevalence of 9.1%, with rates of 4.9% in randomized trials and 17% in cohort studies [6, 7]. The most common adverse reactions include statin‐associated muscle symptoms, such as myalgia, myopathy, myositis with elevated creatine kinase, and rhabdomyolysis [3]. Reported adverse effects of statins also include elevations in hepatic transaminases, while clinically significant myopathy, rhabdomyolysis, or hepatotoxicity occur rarely. Concerns regarding neurocognitive, neurological, renal, gastrointestinal, and reproductive effects have also been reported, although causal evidence for many of these associations remains limited or inconsistent. Statin‐associated muscle symptoms are the most frequently reported adverse effects [4, 7, 8, 9, 10].

In this case, cTnI and CK‐MB were within their reference ranges, whereas myoglobin was elevated. Myocardial injury is defined by at least one cardiac troponin value above the 99th‐percentile upper reference limit, and the injury is considered acute when a rise and/or fall in troponin values is observed [11, 12, 13]. Myoglobin is not cardiac‐specific because it is present in both cardiac and skeletal muscle, and its diagnostic specificity is further reduced in patients with skeletal‐muscle injury or renal dysfunction [13]. Accordingly, the normal cTnI and CK‐MB levels in this patient do not support acute myocardial injury or myocardial necrosis. The elevated myoglobin level may instead reflect skeletal‐muscle release and/or impaired renal clearance. The bradyarrhythmia should therefore be interpreted in the broader context of severe bradycardia, electrolyte imbalance, systemic inflammation, and acute hepatic and renal dysfunction.

NT‐proBNP was elevated to 1912 ng/L. This finding was considered multifactorial rather than diagnostic of overt heart failure. Renal dysfunction substantially affects natriuretic‐peptide concentrations through impaired clearance and its association with volume and cardiovascular stress [14, 15], while profound bradyarrhythmia may also contribute to transient myocardial wall stress. The patient had no reported congestion and echocardiography showed no structural abnormality or systolic dysfunction; therefore, the available data did not support a diagnosis of decompensated heart failure.

Potential mechanisms linking high‐dose statin exposure and systemic organ injury include altered mitochondrial metabolism, oxidative stress, and disturbed cellular energy homeostasis, but much of the mechanistic evidence is preclinical and cannot prove causality in this patient [16]. Pharmacokinetic studies have demonstrated ethnic differences in systemic exposure to some statins, including higher rosuvastatin exposure in Asian participants, although these findings cannot be directly extrapolated to atorvastatin in this patient [17]. Chinese expert guidance therefore emphasizes individualized statin dosing and careful assessment of adverse effects [18]. In the present case, hepatic and renal dysfunction, hyponatremia, hypochloremia, hypocalcemia, inflammation, and metabolic stress may together have lowered the threshold for sinus node dysfunction.

Persistent symptomatic sinus arrest after correction of reversible abnormalities justified permanent pacing according to guideline principles that emphasize correlation between symptoms and sinus node dysfunction rather than a single heart‐rate threshold [19]. The subsequent decrease in pacing burden from approximately 80% to 20% after reducing the LRL from 70 to 50 beats/min is important: it shows partial intrinsic recovery and illustrates that pacing burden depends on device programming. Randomized data in patients with sinus node dysfunction likewise demonstrate that programming a lower base rate markedly reduces atrial pacing, although excessively low‐rate programming may increase presyncope or syncope [20]. Thus, the follow‐up supports persistent or intermittent—not necessarily irreversible—sinus node dysfunction.

Consequently, this case should not be construed as definitive evidence of irreversible statin‐induced sinus node disease. Rather, it supports the hypothesis that high‐dose atorvastatin exposure, compounded by subsequent hepatic and renal impairment, electrolyte disturbances, inflammation, and metabolic derangements, may have contributed to clinically significant, albeit partially reversible, sinus node dysfunction.

Several limitations should be acknowledged. This is a single retrospective case; serum atorvastatin concentrations were unavailable, and a formal drug‐rechallenge was neither performed nor ethically appropriate. Cardiac magnetic resonance imaging, Lyme serology, electrophysiologic testing, and genomic or pharmacokinetic studies were not available. In addition, simultaneous organ dysfunction and electrolyte abnormalities are major confounders. The report therefore supports a possible, indirect, and multifactorial atorvastatin‐associated event rather than definitive direct statin‐induced sinus node disease.

Collectively, the temporal correlation among unsupervised high‐dose atorvastatin use, acute multiorgan dysfunction, severe bradyarrhythmia, and subsequent partial rhythm recovery suggests a potential atorvastatin‐associated adverse event. Nevertheless, the residual pacing requirement and the potential confounding effects of renal dysfunction, electrolyte imbalances, and systemic metabolic abnormalities imply an indirect, multifactorial pathogenesis rather than a direct drug toxicity. Further investigations are warranted to elucidate whether statins can precipitate clinically significant conduction abnormalities in susceptible individuals, particularly in the context of high‐dose exposure and acute organ dysfunction.

## Author Contributions

**Xiao‐Qing Kou:** conceptualization, data curation, formal analysis, investigation, methodology, writing – original draft, writing – review and editing. **Yan‐Zhen Wang:** conceptualization, funding acquisition, investigation, resources, supervision, writing – review and editing. **Yi‐Rong Gan:** investigation, methodology, resources, writing – review and editing. **Yun‐Long Zhang:** investigation, resources. **Shu‐Yun He:** data curation, formal analysis, investigation. **Zheng Zhang:** funding acquisition, supervision, writing – review and editing.

## Funding

This work was supported by Lanzhou Science and Technology Plan Project, 2025‐2‐142. Natural Science Foundation of Gansu Province, 25JRRA1043.

## Ethics Statement

The study followed the principles of the Ethics Committee of Lanzhou First People's Hospital, and the patient was informed of his rights and risks and signed an informed consent form agreeing to participate in the study.

## Consent

Written informed consent was obtained from the patient for publication of this case report and any accompanying images.

## Conflicts of Interest

The authors declare no conflicts of interest.

## Data Availability

All data generated during this study are included in this published article.

## References

[1] R. S. Blumenthal , P. B. Morris , M. Gaudino , et al., “2026ACC/AHA/AACVPR/ABC/ACPM/ADA/AGS/APhA/ASPC/ NLA/PCNA Guideline on the Management of Dyslipidemia: A Report of the American College of Cardiology/American Heart Association Joint Committee on Clinical Practice Guidelines,” Journal of the American College of Cardiology 87 (2026): 2624–2757, 10.1016/j.jacc.2025.11.016. 41824590

[2] R. Chou , A. Cantor , T. Dana , et al., “Statin Use for the Primary Prevention of Cardiovascular Disease in Adults: Updated Evidence Report and Systematic Review for the US Preventive Services Task Force,” JAMA 328 (2022): 754–771, 10.1001/jama.2022.12138. 35997724

[3] Y. Tsushima and B. Hatipoglu , “Statin Intolerance: A Review and Update,” Endocrine Practice 29 (2023): 566–571, 10.1016/j.eprac.2023.03.004. 36958647

[4] C. B. Newman , D. Preiss , J. A. Tobert , et al., “Statin Safety and Associated Adverse Events: A Scientific Statement From the American Heart Association,” Arteriosclerosis, Thrombosis, and Vascular Biology 39 (2019): e38–e81, 10.1161/atv.0000000000000073. 30580575

[5] C. Baigent , L. Blackwell , J. Emberson , et al., “Efficacy and Safety of More Intensive Lowering of LDL Cholesterol: A Meta‐Analysis of Data From 170,000 Participants in 26 Randomised Trials,” Lancet 376 (2010): 1670–1681, 10.1016/s0140-6736(10)61350-5. 21067804 PMC2988224

[6] I. Bytyçi , P. E. Penson , D. P. Mikhailidis , et al., “Prevalence of Statin Intolerance: A Meta‐Analysis,” European Heart Journal 43 (2022): 3213–3223, 10.1093/eurheartj/ehac015. 35169843 PMC9757867

[7] M. K. Cheeley , J. J. Saseen , A. Agarwala , et al., “NLA Scientific Statement on Statin Intolerance: A New Definition and Key Considerations for ASCVD Risk Reduction in the Statin Intolerant Patient,” Journal of Clinical Lipidology 16 (2022): 361–375, 10.1016/j.jacl.2022.05.068. 35718660

[8] M. S. Jeeyavudeen , J. M. Pappachan , and G. Arunagirinathan , “Statin‐Related Muscle Toxicity: An Evidence‐Based Review,” touchREVIEWS in Endocrinology 18 (2022): 89–95, 10.17925/ee.2022.18.2.89. 36694885 PMC9835810

[9] W. H. Kim , C. H. Lee , J. H. Han , et al., “C/EBP Homologous Protein Deficiency Inhibits Statin‐Induced Myotoxicity,” Biochemical and Biophysical Research Communications 508 (2019): 857–863, 10.1016/j.bbrc.2018.11.166. 30528737

[10] A. Muñoz‐Blanco , R. Gómez‐Huelgas , and J. F. Gómez‐Cerezo , “Statin‐Associated Muscle Symptoms: Myth or Reality?,” Rev Clin Esp (Barc) 222 (2022): 602–611, 10.1016/j.rceng.2022.03.006. 35810133

[11] D. Brieger , L. Cullen , T. Briffa , et al., “National Heart Foundation of Australia & Cardiac Society of Australia and New Zealand: Comprehensive Australian Clinical Guideline for Diagnosing and Managing Acute Coronary Syndromes 2025,” Heart, Lung & Circulation 34 (2025): 309–397, 10.1016/j.hlc.2025.02.102. 40180468

[12] S. V. Rao , M. L. O'Donoghue , M. Ruel , et al., “2025 ACC/AHA/ACEP/NAEMSP/SCAI Guideline for the Management of Patients With Acute Coronary Syndromes: A Report of the American College of Cardiology/American Heart Association Joint Committee on Clinical Practice Guidelines,” Circulation 151 (2025): e771–e862, 10.1161/cir.0000000000001309. 40014670

[13] J. McCord , R. M. Nowak , P. A. McCullough , et al., “Ninety‐Minute Exclusion of Acute Myocardial Infarction by Use of Quantitative Point‐Of‐Care Testing of Myoglobin and Troponin I,” Circulation 104 (2001): 1483–1488, 10.1161/hc3801.096336. 11571240

[14] B. L. Neuen , M. Vaduganathan , B. L. Claggett , et al., “Natriuretic Peptides, Kidney Function, and Clinical Outcomes in Heart Failure With Preserved Ejection Fraction,” JACC Heart Fail 13 (2025): 28–39, 10.1016/j.jchf.2024.08.009. 39365237

[15] M. Nyberg , D. Terzic , T. P. Ludvigsen , et al., “A State of Natriuretic Peptide Deficiency,” Endocrine Reviews 44 (2023): 379–392, 10.1210/endrev/bnac029. 36346821 PMC10166265

[16] A. Liu , Q. Wu , J. Guo , et al., “Statins: Adverse Reactions, Oxidative Stress and Metabolic Interactions,” Pharmacology & Therapeutics 195 (2019): 54–84, 10.1016/j.pharmthera.2018.10.004. 30321555

[17] E. Lee , S. Ryan , B. Birmingham , et al., “Rosuvastatin Pharmacokinetics and Pharmacogenetics in White and Asian Subjects Residing in the Same Environment,” Clinical Pharmacology & Therapeutics 78 (2005): 330–341, 10.1016/j.clpt.2005.06.013. 16198652

[18] J. J. Li , K. F. Dou , Z. G. Zhou , et al., “Chinese Expert Consensus on the Clinical Diagnosis and Management of Statin Intolerance,” Clinical Pharmacology and Therapeutics 115 (2024): 954–964, 10.1002/cpt.3213. 38459425

[19] F. M. Kusumoto , M. H. Schoenfeld , C. Barrett , et al., “2018 ACC/AHA/HRS Guideline on the Evaluation and Management of Patients With Bradycardia and Cardiac Conduction Delay: A Report of the American College of Cardiology/American Heart Association Task Force on Clinical Practice Guidelines and the Heart Rhythm Society,” Journal of the American College of Cardiology 74 (2019): e51–e156, 10.1016/j.jacc.2018.10.044. 30412709

[20] M. B. Kronborg , M. H. J. P. Frausing , J. Malczynski , et al., “Atrial Pacing Minimization in Sinus Node Dysfunction and Risk of Incident Atrial Fibrillation: A Randomized Trial,” European Heart Journal 44 (2023): 4246–4255, 10.1093/eurheartj/ehad564. 37638973 PMC10590128

